# Multiscale Three-Dimensional Vertical Graphene-Encapsulated Nanoparticle Coatings for Antibacterial Applications against Multidrug-Resistant Bacteria

**DOI:** 10.1021/acsanm.5c05084

**Published:** 2026-01-01

**Authors:** Jian Zhang, Santosh Pandit, Shadi Rahimi, Zhejian Cao, Ivan Mijakovic

**Affiliations:** † Systems and Synthetic Biology Division, Department of Life Sciences, 11248 Chalmers University of Technology, SE-412 96 Gothenburg, Sweden; ‡ The Novo Nordisk Foundation, Center for Biosustainability, Technical University of Denmark, DK-2800 Kongens Lyngby, Denmark

**Keywords:** 3D coating, vertical graphene, nanoparticles, drug-resistant bacteria, antibacterial activity

## Abstract

Implant-associated infections caused by multidrug-resistant *Staphylococcus aureus* (*S. aureus*) remain a major clinical challenge, underscoring the urgent need for surface-engineered antibacterial strategies that extend beyond conventional antibiotic delivery. Here, we report a hierarchical antimicrobial coating that integrates chemical surface functionalization, nanoparticle (NP) assembly, vertical graphene (VG) growth, and antibiotic incorporation into a hybrid platform. The resulting three-dimensional composite coating (Si/APTES/NPs/VG) features a NP-mediated interfacial layer and vertically oriented graphene with a high drug-loading capacity, enabling localized, contact-mediated antibacterial activity. The Si/APTES/NPs/VG/vancomycin coating exhibited potent antibacterial efficacy, achieving a 10000-fold reduction in viable bacteria, achieving 99.99% antibacterial efficiency. Importantly, the coating also maintained favorable cytocompatibility, highlighting its potential for biomedical implant applications. Overall, this work establishes a versatile strategy for constructing carbon-based bioactive surfaces and provides a promising approach for preventing infections caused by drug-resistant *S. aureus*.

## Graphical abstract

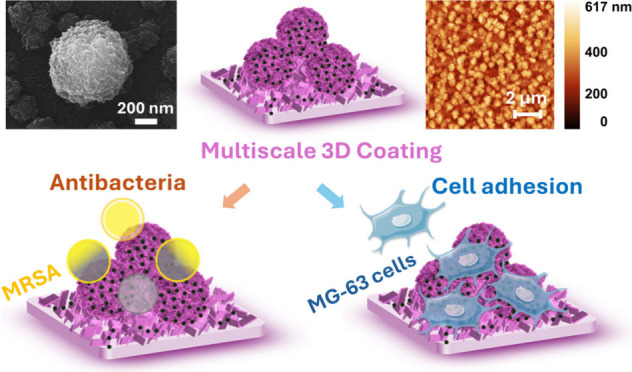

The global emergence of multidrug-resistant bacteria, particularly methicillin-resistant *Staphylococcus aureus* (MRSA), has become a major clinical challenge.^1^ MRSA infections exhibit a wide spectrum of clinical manifestations, ranging from skin and soft tissue infections to severe and potentially life-threatening diseases such as sepsis. Effective prevention of surgical site infections remains a critical challenge, particularly in orthopedic procedures.^2^ MRSA can form robust biofilms on implant surfaces, which significantly increases bacterial tolerance to antibiotics, requiring concentrations far higher than those needed to eliminate planktonic bacteria. Orthopedic implant-associated infections involving biofilms are difficult to treat using conventional methods, often resulting in delayed recovery, reduced patient quality of life, and substantial economic burden.^3,4^ With the growing global use of implants, preventing biofilm formation has become an urgent priority. There is a critical need for novel antimicrobial materials and coatings that can both prevent biofilm-related infections and circumvent antibiotic resistance.^5^ Nanoparticle (NP)-based antibacterial strategies have attracted increasing attention due to their ability to integrate multiple therapeutic modalities.^6–8^ For example, engineered piezoelectric hydrogels incorporating doped BaTiO_3_ NPs can generate reactive oxygen species (ROS) under ultrasound stimulation, modulate immune responses, and effectively suppress MRSA biofilms, demonstrating the power of nanoscale engineering to enhance antimicrobial performance.^9^ These advances highlight the importance of rational nanoscale design in developing multifunctional antibacterial systems. In addition, surface nanoengineering has emerged as a promising route for preventing biofilm formation. Antibacterial surfaces could effectively inhibit biofilm formation through mechanisms such as physical membrane disruption, modulation of wettability, charge-mediated interactions, or serving as platforms for drug delivery.^10^ For example, graphene oxide, due to its abundance of oxygen-containing groups and large surface area, has been explored as a drug loading carrier.^11^ Metal–organic framework-based antibacterial layers can physically damage bacteria like tiny spikes, directly piercing and killing them.^12^

Among various nanostructured coatings, vertically oriented graphene has attracted particular interest over recent years.^13^ Vertical graphene (VG) synthesized via plasma-enhanced chemical vapor deposition (PECVD) is considered as a promising candidate due to its unique mechanical robustness, chemical stability, and vertically aligned nanoflakes architecture.^14,15^ Composed of graphene nanoflake arrays standing perpendicular to the substrate, VG coatings present exposed sharp edges that can physically disrupt bacterial membranes upon contact. This provides potent contact-based antimicrobial mechanism that remains effective regardless of antimicrobial resistance.^16–18^ Moreover, VG has been reported to exhibit photothermal properties, enabling rapid bacterial inactivation under near-infrared irradiation.^19^ Most reported VG coatings have been developed on uniform, flat substrates such as silicon wafers or titanium. It has been observed that electric fields may influence VG growth, which tends to occur preferentially in regions of locally enhanced field strength.^20–22^ On the NP surface, VG grows along the normal direction of the surface, which is consistent with the local electric field.^23^

Here, we report a novel three-dimensional (3D) antibacterial coating for combating infections caused by drug-resistant *Staphylococcus aureus*. This study proposes a multilevel bottom-up strategy to construct multiscale coatings through sequential surface functionalization, NPs self-assembly, vertical graphene growth, and drug loading (Figure 1). A Si wafer was first modified with (3-aminopropyl)­triethoxysilane (APTES) to guide the electrostatic assembly of carboxyl-functionalized NPs. The NPs layer served as a template and interfacial platform for subsequent PECVD growth of VG nanoflakes (Si/APTES/NPs/VG). Vancomycin was finally loaded to form a 3D composite coating (Si/APTES/NPs/VG/Van) for combating drug-resistant *S. aureus*. Unlike previously reported VG antimicrobial coatings grown on planar substrates, this study employs a NPs interfacial layer to direct VG growth, thereby constructing a 3D hierarchical architecture. This multiscale morphology not only preserves the characteristic edge-rich features of VG but also substantially increases the effective surface area available for molecular interactions, providing favorable conditions for antibiotic immobilization. Within this hierarchical structure, vancomycin can be efficiently immobilized and locally presented, thereby extending VG-based antimicrobial surfaces from systems that rely primarily on contact-mediated interactions to multifunctional antimicrobial platforms incorporating drug-assisted enhanced bioactivity. To the best of our knowledge, there have been no prior reports on the growth of VG on NPs interfacial layers for antimicrobial applications. This work demonstrates that NPs-guided VG offers a structural pathway for integrating drug loading into VG antimicrobial coatings, thereby broadening their application potential in the prevention of implant-associated infections.

**Figure 1:**
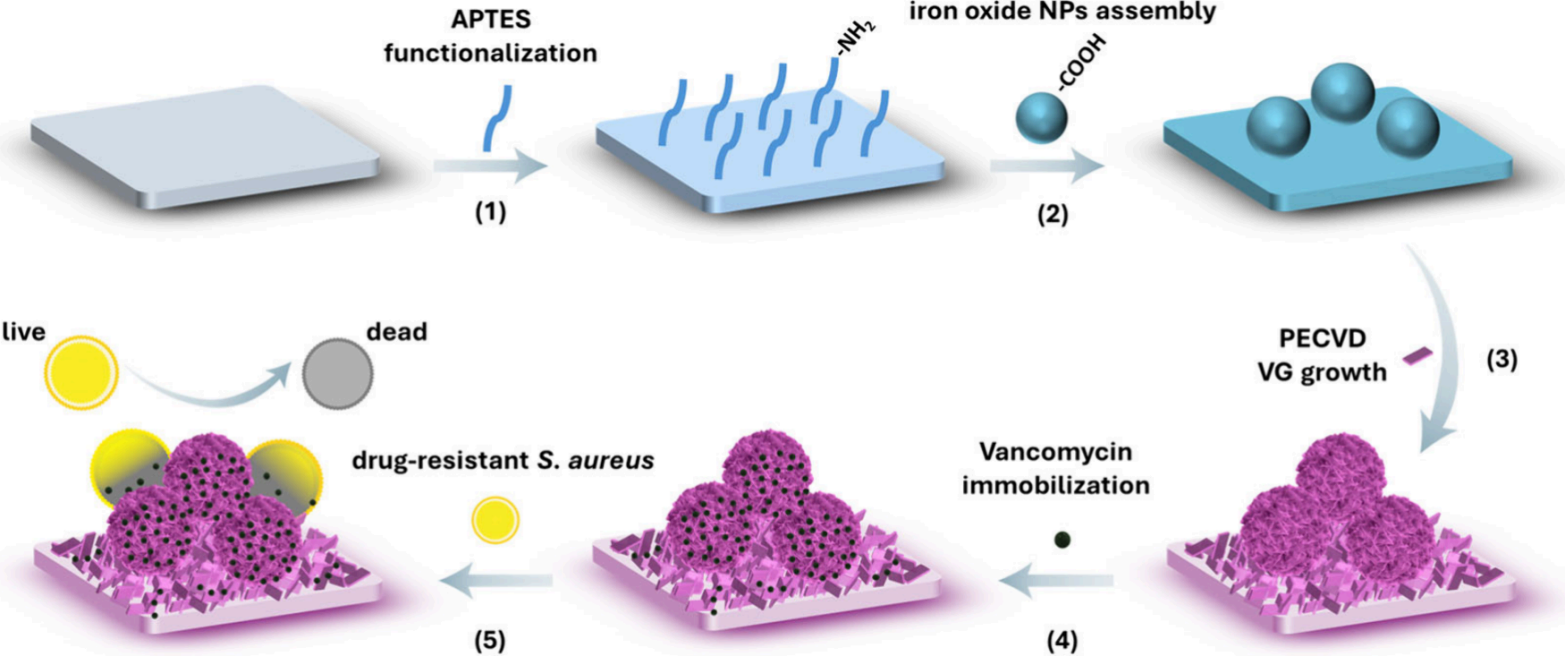
Schematic diagram of 3D Si/APTES/NPs/VG/Van coatings for antibacterial application. (1) Surface functionalization: The Si wafer was modified with APTES to introduce positively charged amino groups; (2) NPs assembly: Carboxyl-functionalized iron oxide NPs were electrostatically assembled onto the Si/APTES surface; (3) VG growth: The Si/APTES/NPs layer served as an interfacial platform for VG growth by PECVD; (4) Antibiotic loading: Vancomycin was loaded into Si/APTES/NPs/VG coating; (5) Antibacterial: The Si/APTES/NPs/VG/van coating was applied for combating drug-resistant *S. aureus*.

Vancomycin-loaded VG-encapsulated NP (Si/APTES/NPs/VG/Van) coating was fabricated as described in the experimental section in the Supporting Information. Carboxyl-functionalized iron oxide NPs were electrostatically assembled onto Si/APTES wafer, producing a uniform monolayer (Figure 2A). VG were subsequently grown on this NP layer via PECVD, yielding a network of graphene nanoflakes with sharp edges characteristic of VG architectures (Figure 2B). The NPs retained a protruding topography relative to the substrate background. On almost round-shaped NPs, VG grows along the field direction, that is, perpendicular to the particle surface (Figure 2C). Additional SEM images of Si/APTES/NPs and Si/APTES/NPs/VG coatings are shown in Figure S1. Energy dispersive spectroscopy (EDS) mapping and spectra of the Si/APTES/NPs/VG/Van coatings are shown in Figure S2. While AFM images of Si/APTES/NPs (Figure 2D) and Si/APTES/NPs/VG/Van (Figure 2E) showed the morphologies consistent with the SEM observations. Raman spectroscopy was used to examine the structure of the composite coatings. The Si/APTES exhibited a typical Raman spectrum, with a prominent peak at 519 cm^–1^ and broad features between 900 and 1000 cm^–1^ corresponding to first and second-order silicon scattering, respectively (Figure 2F, curve a).^24^ Upon NPs assembly, additional Raman features appeared at 217, 280, 392, and 590 cm^–1^, corresponding respectively to the A_1_g symmetric stretching of the Fe–O framework in octahedra, the E_1_g in-plane bending of oxygen atoms, the E_1_g in-plane vibration associated with Fe–O–Fe bond angle variations, and the high-frequency A_1_g symmetric stretching of strong Fe–O bonds in α-Fe_2_O_3_ (Figure 2F, curve b).^25^ Following VG growth, the Raman spectrum was dominated by the characteristic D (1350 cm^–1^), G (1590 cm^–1^), and 2D (2650 cm^–1^) bands of graphene (Figure 2F, curve c). The D band arises from defect- and edge-activated scattering, whereas G band corresponds to the in-plane stretching mode of sp^2^-hybridized C–C bonds. The intensity ratio *I*_D_/*I*_G_ is widely used as an indicator of defect density and edge abundance in graphene, with higher values reflecting increased structural disorder.^26,27^ The ID/IG value of 1.17 obtained for our samples indicates a high density of edges and defects, which is characteristic of plasma-grown vertical graphene (VG). In addition, the 2D band is weak and broadened, consistent with multilayer graphene stacking commonly observed in VG.^23^ These Raman features confirm that the coating is composed of graphene rich in edges and defects, which is the characteristic of VG structure. The morphology observed by SEM, together with the Raman spectral signatures, is consistent with previously reported NPs-assisted vertical graphene growth in PECVD systems.^23^ On NPs, VG does not grow strictly perpendicular to the substrate; instead, it follows the local electric-field direction and grows perpendicular to the NPs’ local surfaces.

**Figure 2:**
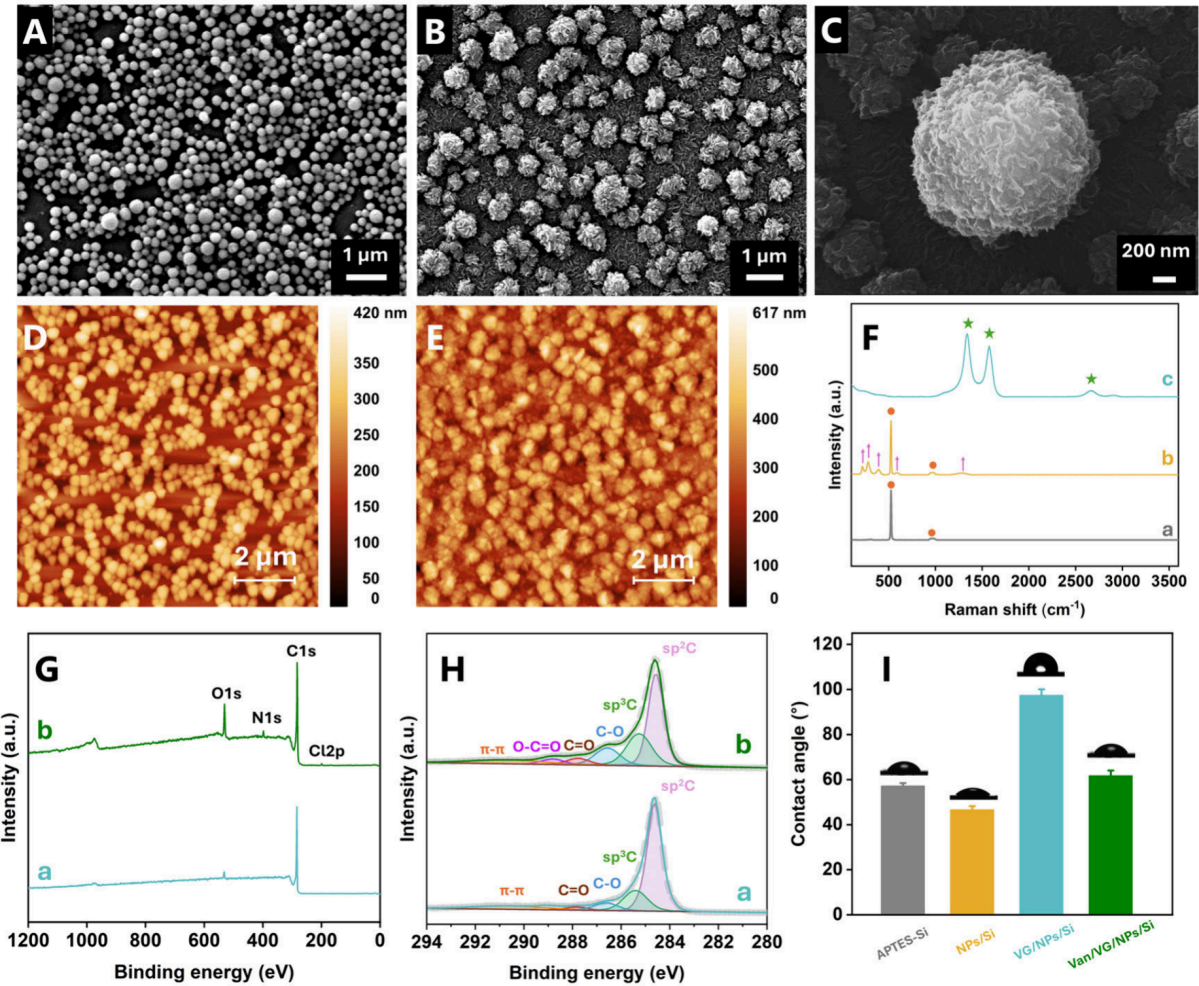
Surface characterization. (A–C) SEM images of groups b (A) and c (B, C); (D,E) AFM images of groups b (D) and c I (E); (F) Raman spectra of groups a–c; (G) Full-scan XPS spectra of groups c (a) and d (b); (H) High-resolution C 1s spectra of groups c (a) and d (b); (I) Water contact angles of groups a–d. Groups: (a) Si/APTES; (b) Si/APTES/NPs; (c) Si/APTES/NPs/VG; (d) Si/APTES/NPs/VG/Van.

The surface chemical compositions of the Si/APTES/NPs/VG and Si/APTES/NPs/VG/Van coatings were analyzed by X-ray photoelectron spectroscopy (XPS). The survey spectrum (Figure 2G) reveals distinct differences in elemental composition between the two surfaces, with the corresponding atomic percentages summarized in Table S1. The Si/APTES/NPs/VG coating is dominated by carbon, with a minor oxygen contribution attributable to adventitious surface contamination. In contrast, the Si/APTES/NPs/VG/Van coating exhibits a pronounced increase in the O 1s signal, accompanied by the emergence of distinct N 1s and Cl 2p peaks, which are characteristic elements of vancomycin, confirming its successful immobilization on the VG surface. High-resolution C 1s spectra provides further insight into the chemical state changes induced by vancomycin loading. For the Si/APTES/NPs/VG coating (Figure 2H, curve a), the C 1s spectrum was deconvoluted into two dominant components at 284.5 and 285.7 eV, corresponding to sp^2^- and sp^3^-hybridized carbon, respectively, along with minor contributions from oxygen-containing groups, including C–O (286.9 eV) and C = O (287.8 eV).^28^ Upon vancomycin loading (Figure 2H, curve b), additional components appear at 286.2, 287.8, 288.9, and 290.9 eV, which are assigned to C–O, C=O, O–C=O, and π–π* interactions, respectively. The increased contribution of oxygen-containing carbon species reflects the presence of multiple hydroxyl, carboxyl, and aromatic functional groups inherent to vancomycin, supporting its adsorption onto the VG surface.^29^ High-resolution O 1s, N 1s, and Cl 2p spectra further corroborate these assignments and are provided in Figure S3. In addition, Fourier-transform infrared (FTIR) spectroscopy was performed to probe vancomycin immobilization. However, due to the strong infrared absorption background from the silicon substrate and the VG layer, the characteristic vibrational bands of vancomycin were significantly attenuated and could not be clearly resolved. Overall, the XPS results provide compelling evidence of vancomycin loading, as indicated by the appearance of N 1s and Cl 2p signals and the increased contribution of oxygen-containing components in the C 1s spectrum following vancomycin immobilization. The wettability of the coatings was evaluated via water contact angle measurements (Figure 2I). The Si/APTES exhibited a contact angle of 58°. Following NP assembly, the contact angle of Si/APTES/NPs decreased, consistent with hydrophilic surface functionalities on the carboxyl-functionalized NPs.^30^ However, Si/APTES/NPs/VG displayed pronounced hydrophobicity (95°), in agreement with prior reports for VG.^18^ Upon vancomycin immobilization, the contact angle of the Si/APTES/NPs/VG/Van decreased substantially, showing a transition from hydrophobic to more hydrophilic character. The enhanced wettability may be attributed to the high density of polar groups (e.g., hydroxyl and amino moieties) in vancomycin, further supporting its successful immobilization on the VG surface.^31^

Biofilms can produce extracellular matrixes to encapsulate bacterial population, thereby enhancing antibiotic resistance and posing a significant challenge to the management of bacterial infections.^32^ Here, we examined the antibiofilm activity of the composite coatings using representative Gram-positive bacterium, the multidrug-resistant *S. aureus* CCUG35571. As shown in Figure 3A, Si/APTES/NPs/Van and Si/APTES/NPs/VG/Van showed significantly reduced biofilm growth from crystal violet staining. Si/APTES/NPs/Van exhibited a reduced OD_590_ value, indicating an inhibitory effect on biofilm formation, attributed to the presence of vancomycin loaded on the NPs. Moreover, the OD_590_ value of the Si/APTES/NPs/VG/Van was significantly reduced, indicating a strong inhibitory effect on biofilm formation. This may be due to the high vancomycin loading capacity of the VG surface compared to the NPs. Furthermore, the number of viable bacteria in the residual biofilm was determined by colony counting. As shown in Figure 3B, the CFU counts of the Si/APTES/NPs/Van and Si/APTES/NPs/VG/Van were reduced by 100 and 10000-fold, respectively, compared to the Si control. Both groups exhibited a significant reduction in viable bacterial viability, where efficiency of Si/APTES/NPs/VG/Van group found to be higher compared to other counterparts. Live/dead fluorescence staining, as shown in Figure 3C, further confirmed the effectiveness of Si/APTES/NPs/VG/Van in preventing bacterial colonization and biofilm formation. No dead cells were observed on the Si control. Similarly, the Si/APTES/Van showed almost no loss of bacterial adhesion, which may be due to the inefficient loading of vancomycin. In contrast, the Si/APTES/NPs/Van sample exhibited a reduction in viable cells. Notably, the viable bacteria on the Si/APTES/NPs/VG/Van coating were greatly reduced, suggesting more effective in preventing bacterial surface colonization. Furthermore, changes in bacterial morphology and biofilm organization on different surfaces were examined by SEM (Figure 3D). Consistent with the results from live/dead fluorescence imaging, dense and continuous biofilm structures were observed on the bare Si and Si/APTES/Van surfaces. In contrast, the bacterial density on the Si/APTES/NPs/Van surface was markedly reduced, while only sparse bacterial cells were detected on the Si/APTES/NPs/VG/Van surface, indicating a pronounced suppression of biofilm formation. These observations demonstrate that the Si/APTES/NPs/VG/Van surface exhibits a pronounced inhibitory effect on biofilm formation within 24 h, achieving a 99.99% antibacterial efficacy. To further evaluate whether the antibacterial activity of the Si/APTES/NPs/VG/Van coating persists over extended periods, antibacterial assessments were conducted for up to 3 days. Viable bacterial counts were recorded daily, with the culture medium replaced every 24 h, and the results are presented in Figure S4. CFU analysis demonstrated that the Si/APTES/NPs/VG/Van surface retained antibacterial activity after 3 days of biofilm culture, resulting in an approximately 90% reduction in viable cell counts. These results indicate that the Si/APTES/NPs/VG/Van exhibits significantly enhanced inhibition of biofilm maturation and bacterial recolonization compared with the Si/APTES/Van and Si/APTES/NPs/Van, thereby highlighting its enhanced antibacterial performance. This enhanced activity is likely associated with the higher vancomycin loading enabled by the hierarchical VG architecture. To elucidate the contribution of vancomycin, antibiotic loading on different coatings were evaluated (Figure S5). The Si/APTES surface exhibited negligible vancomycin adsorption, whereas higher loading was achieved on Si/APTES/NPs. The highest loading was observed for the Si/APTES/NPs/VG surface, demonstrating that the NPs-guided VG architecture provides additional accessible surface area and interaction sites for antibiotic immobilization.^33^ Notably, no quantifiable vancomycin release was detected by UV–vis spectroscopy at 280 nm. This observation is likely attributable to the limited sensitivity of the used technique. Vancomycin association with the VG surface is primarily governed by noncovalent interactions, including π–π stacking and hydrogen bonding, which stabilizes antibiotic at the graphene interface.^29,34,35^ Despite the absence of a quantifiable vancomycin signal in the bulk medium, the Si/APTES/NPs/VG/Van coating exhibited enhanced antibacterial activity, suggesting that surface-loaded vancomycin enables the establishment of a locally enriched antibiotic microenvironment at the bacteria–material interface to inhibit bacterial colonization and biofilm formation.

**Figure 3:**
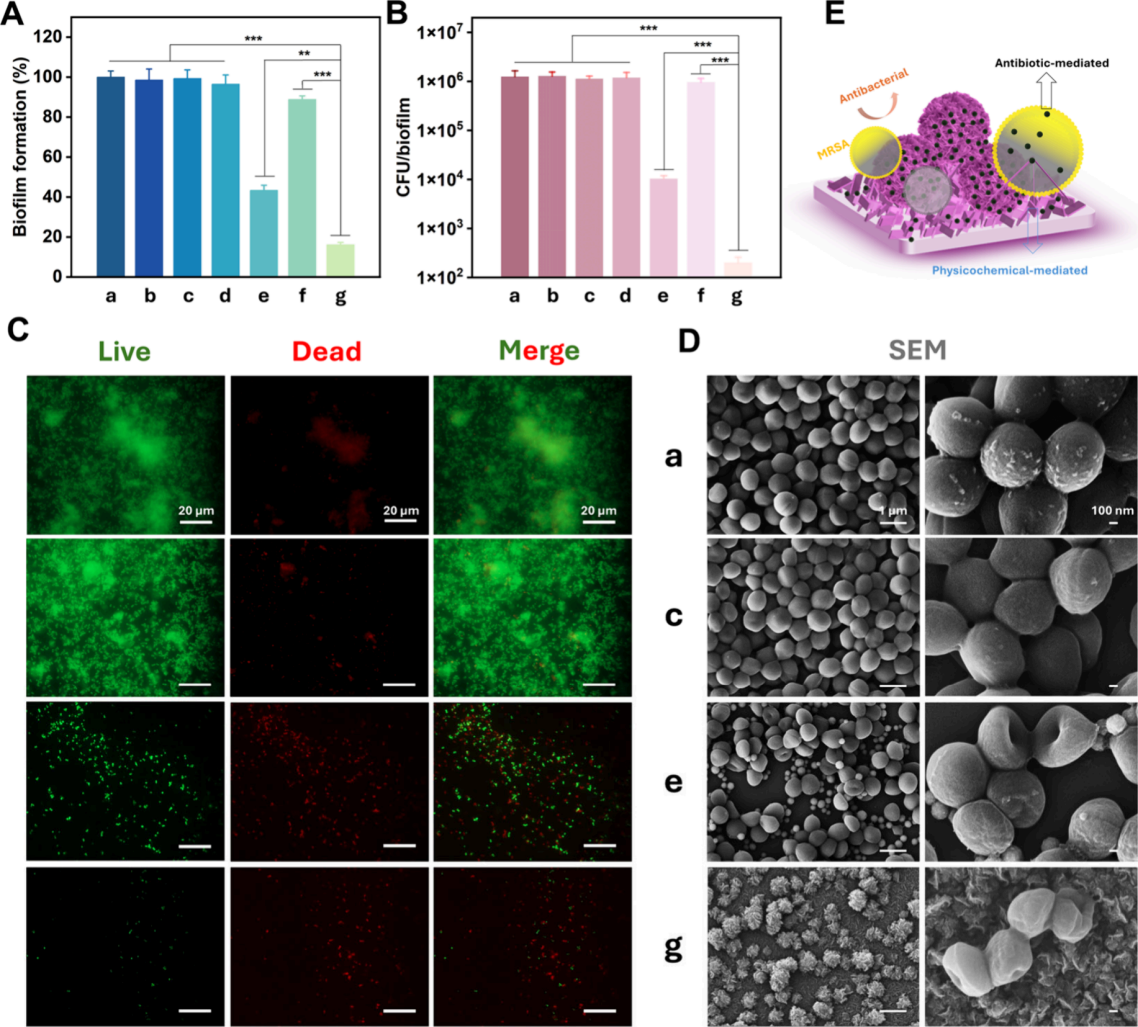
Antibacterial performance. (A) Inhibition rate of biofilm formation measured by crystal violet (CV) staining for groups a-g; (B) Viable bacterial count determined by CFU assay for groups a-g; (C) Live/Dead staining images showing bacterial viability for groups (a, c, e, g); (D) SEM images of biofilm morphology for groups (a, c, e, g). Groups: (a) bare Si; (b) Si/APTES; (c) Si/APTES/Van; (d) Si/APTES/NPs; (e) Si/APTES/NPs/Van; (f) Si/APTES/NPs/VG; (g) Si/APTES/NPs/VG/Van; (E) Schematic illustration of Si/APTES/NPs/VG/Van coatings against drug-resistant *S. aureus*. Data represent the mean ± standard deviation of three biological replicates (*p < 0.05, **p < 0.01, ***p < 0.001).

Previous mechanistic studies have demonstrated that the sharp edges of graphene play a critical role in their antibacterial activity.^36,37^ On one hand, graphene edges can physically damage bacterial cell membranes by extracting lipid molecules or perforating the membrane structure.^38^ On the other hand, structural defects at graphene edges can induce oxidative damage to cell membranes or intracellular components through the generation of reactive oxygen species (ROS) or via direct electron transfer. These processes lead to oxidative stress in bacterial cells, ultimately resulting in cell death.^39^ In this study, we observed pronounced morphological changes in bacterial cells on the unloaded VG surface, including cell flattening, compression, and deformation (Figure S6). These features indicate that the exposed edges of VG nanoflakes induce contact-mediated membrane stress, and that intimate contact with VG can mechanically perturb bacterial membranes. Itis consistent with previous reports on antibacterial interfaces based on VG.^16–19^ In summary, the drug-loading capability of the coating, together with the observed bacterial morphological alterations due to VG, suggests that its antibacterial performance arises from the combined effects of antibiotic-mediated activity and contact-induced membrane stress at the bacteria–material interface (Figure 3E). Although extrapolating in vitro findings to *in vivo* infection scenarios remains challenging, it is noteworthy that the bacterial concentration employed in this study (10^5^ CFU mL^–1^) represents a highly stringent condition, thereby enabling a rigorous evaluation of the antimicrobial performance of the coating.^40^

To evaluate the biocompatibility of the Si/APTES/NPs/VG/Van coating, the MG-63 human osteoblast cell line was employed as a model. Figure 4A presents the results of a standard alamarBlue assay used to assess cell viability after 24 h exposure to bare Si wafer, Si/APTES/Van, Si/APTES/NPs/Van and Si/APTES/NPs/VG/Van. No statistically significant differences were detected among groups. In addition, morphological assessment of cell attachment (Figure 4B) revealed that MG-63 cells exhibited firm anchorage on both Si/APTES/NPs/Van and Si/APTES/NPs/VG/Van surfaces. Cells were uniformly distributed, forming interconnected networks, and displayed a typical polygonal morphology characteristic of well-spread osteoblasts, with abundant filamentous structures and intimate contact with the substrate. Bright-field images (Figure 4C) further confirmed that cells adjacent to the coating maintained well-defined polygonal shapes with extended filopodia, indicative of healthy adhesion and proliferation, thereby demonstrating excellent biocompatibility. Quantitative analysis showed no significant reduction in surface coverage of adhered cells (Figure 4D). Previous studies have reported that VG coatings exhibit high biocompatibility with diverse human and animal cell types, including human adipose-derived stem cells (hADSCs), mouse fibroblasts (NIH3T3), and osteoblasts (MC3T3-E1).^16,17,19^ Nanostructured interfaces represent promising platforms that promote cell proliferation, adhesion, and differentiation. In the present work, the 3D network architecture of the Si/APTES/NPs/VG/Van coating demonstrated outstanding biocompatibility, offering a valuable reference for the rational design of biointerfaces aimed at modulating stem cell fate (Figure 4E).

**Figure 4:**
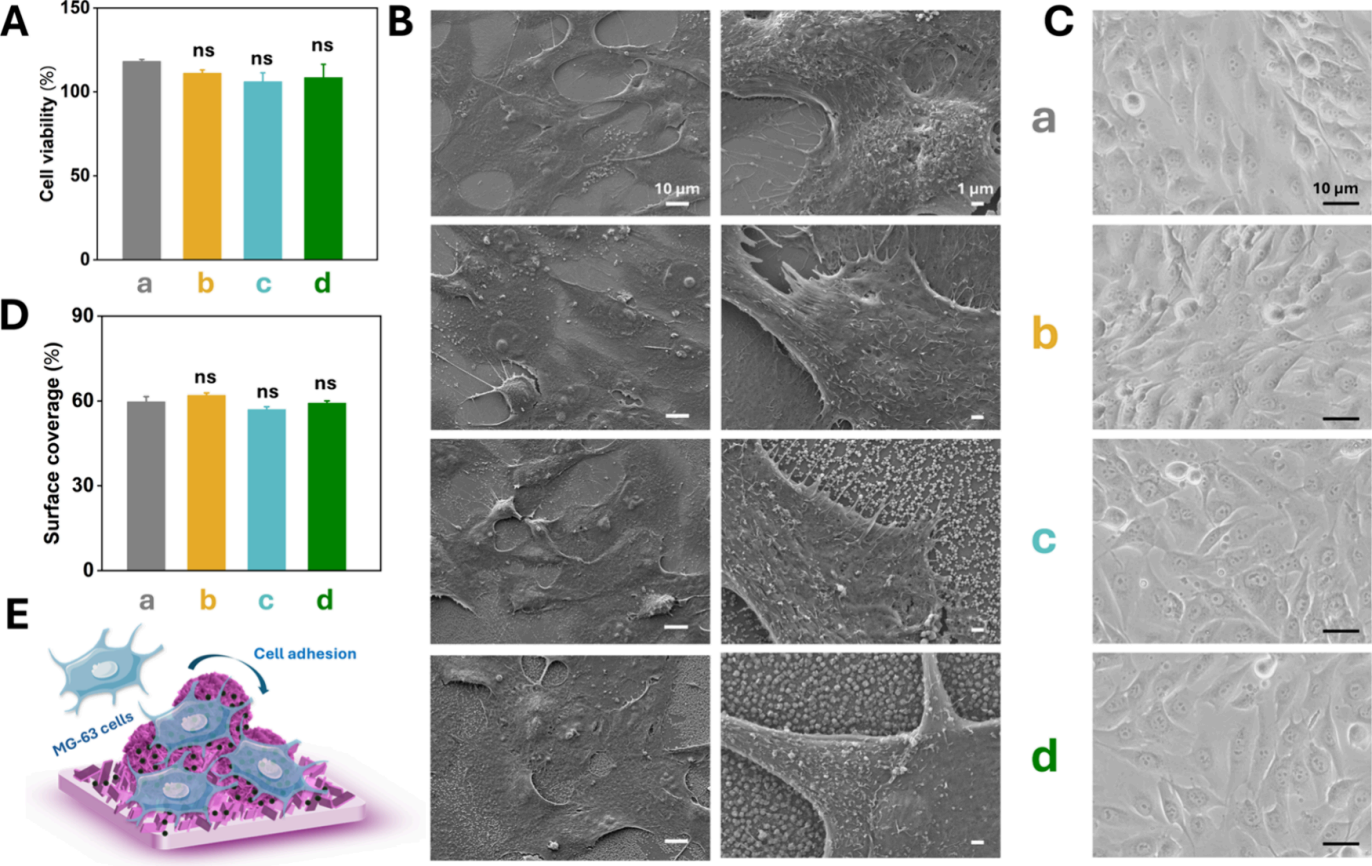
(A) Cell viability of MG-63 cells after culture on different tested sample groups (a–d); (B) SEM and (C) bright-field images of MG-63 cells after culture on different groups (a–d); (D) Surface coverage of MG-63 cells after culture on different groups (a–d); (E) Schematic illustration of MG-63 cells on Si/APTES/NPs/VG/Van coating. (E) Schematic illustration of MG-63 cells cultured on the Si/APTES/NPs/VG/Van coating. Groups: (a) bare Si; (b) Si/APTES/Van; (c) Si/APTES/NPs/Van and (d) Si/APTES/NPs/VG/Van. Data represent the mean ± standard deviation of three biological replicates. No statistically significant differences were found among the four groups (ns, *p* > 0.05).

This study developed a novel antimicrobial coating that is effective against multidrug-resistant *S*. *aureus*. A multiscale bottom-up fabrication strategy was established. The platform was engineered in a stepwise way with surface modification, NPs assembly, VG growth, and drug immobilization. Specifically, APTES was employed to Si wafer enable the self-assembly of negatively charged nanoparticles, which subsequently served as template arrays for VG growth via PECVD. The resulting nanostructured surface was loaded with vancomycin, yielding a 3D composite antimicrobial coating (Si/APTES/NPs/VG/Van). This hierarchical design strategy enables precise control of nanobiointerface engineering and provides a versatile platform for the development of carbon-based bioactive materials to combat implant-associated biofilm infections. Moreover, the antimicrobial activity of this hierarchical nanostructured platform stems from the drug-loading capacity and cellular interactions of VG while maintaining favorable biocompatibility, underscoring the potential of this platform for future *in vivo* and biointegration studies. While this study employs silicon as a model substrate, PECVD-grown VG has been reported on titanium, indicating that the NP-guided VG architecture is potentially transferable to clinically relevant implant materials.^19,41^ This study focused on a drug-resistant *S. aureus* strain as a representative model; future studies will extend antibacterial evaluations to additional *S. aureus* strains as well as multidrug-resistant Gram-negative pathogens to further assess the generality of this coating strategy. Moreover, in *vivo* studies will be essential to evaluate long-term stability, host responses, and infection prevention under physiological conditions. These future efforts will help advance the coating toward translational biomedical applications.

## Supplementary Material



## Notes

Experimental details including strains, chemicals, materials, synthesis of coating, characterizations, antibacterial assays and biocompatibility, SEM images of Si/APTES/NPs and Si/APTES/NPs/VG, EDS mapping images of Si/APTES/NPs/VG, high-resolution O 1s, N 1s, and Cl 2p spectra for Si/APTES/NPs/VG/Van, viability of bacteria in 3 days of biofilms grown on Si/APTES/NPs/VG/Van, amount of vancomycin loading, and SEM images of *S. aureus* after 24 h contact with VG surfaces (PDF)
