# Supplementary material for: Multiscale Three-Dimensional Vertical Graphene-Encapsulated Nanoparticle Coatings for Antibacterial Applications against Multidrug-Resistant Bacteria
Source: ACS Appl Nano Mater. 2026 Jan 1;9(2):921–8. doi: 10.1021/acsanm.5c05084 (PMC12813970; doi:10.1021/acsanm.5c05084)
Supplement: Supplementary file 1 [file an5c05084_si_001.pdf]

Supporting Information

Multiscale Three-Dimensional Vertical Graphene-Encapsulated Nanoparticle Coatings for Antibacterial Applications against Multidrug-Resistant Bacteria

Jian Zhang [a*], Santosh Pandit [a], Shadi Rahimi [a], Zhejian Cao [a], Ivan Mijakovic [a,b*]

[a] Systems and Synthetic Biology Division, Department of Life Sciences, Chalmers University of Technology, SE-412 96 Gothenburg, Sweden

[b] The Novo Nordisk Foundation, Center for Biosustainability, Technical University of Denmark, DK-2800 Kongens Lyngby, Denmark

Corresponding Email: jianzha@chalmers.se; ivan.mijakovic@chalmers.se

KEYWORDS

3D coating, vertical graphene, nanoparticles, drug-resistant bacteria, antibacterial activity

Experimental sections

Bacterial strains, chemicals, and materials

Multidrug-resistant *Staphylococcus aureus* (ccug35571) was obtained from Gothenburg University Culture Collection (CCUG) and used for evaluation of antibacterial activity. Tryptic soy broth (TSB) broth was bought from Sigma-Aldrich (Sweden). Vancomycin hydrochloride and (3-Aminopropyl)triethoxysilane (APTES) were purchased from Sigma-Aldrich (Sweden). All the chemicals were used without further purification. Carboxylate nanoparticles (300 nm diameter, product code: 02131) were purchased from Ademtech (France). The nanoparticles exhibit a polydispersity index (PDI) of 0.09 and a negative surface charge, with a zeta potential of approximately -25 mV.

Synthesis of Si/APTES/NPs/VG coating

Silicon wafers with 300 nm SiO_2 layer were first cleaned by sequential ultrasonication in acetone, isopropyl alcohol, ethanol, and deionized water for 15 min each. After drying with nitrogen, the substrates were treated with UV-ozone for 30 minutes to remove organic contaminants and introduce surface hydroxyl groups. Subsequently, the activated substrates were immersed in a 2.5% (v/v) APTES solution in anhydrous ethanol for 2 h at room temperature under ambient laboratory humidity, in a covered container. After silanization, the samples were rinsed with ethanol and cured at 110 °C for 1 h to complete the functionalization.

Twenty µL of carboxylate-functionalized nanoparticles dispersion were suspended in 2 ml of PBS-1X adjusted to pH 7.4 in a centrifuge tube. The Si/APTES was maintained vertically in the centrifuge tube fully immersed in the nanoparticles dispersion without stirring at room temperature. The Si/APTES/NPs was rinsed twice with ultrapure water and dried under nitrogen.

VG was grown on the Si/APTES/NPs in a cold-walled low-pressure PECVD reactor (Black Magic, Aixtron). The substrate was heated to 775 °C within 2 min and annealed for 1 min with the mixing of 20 sccm H_2 gas and 1000 sccm Ar gas. Then, the plasma was activated under a DC bias of 75 W. The growth of vertical graphene was initiated by introducing carbon source ethylene (C_2H_2, 15sccm), which was maintained for 5 min. After growth, the system was evacuated to a pressure below 0.2 mbar and subsequently cooled down.

Vancomycin loading and release

Vancomycin was loaded onto the Si/APTES/NPs/VG coatings by immersing each substrate in 1 mL of a 1 mg/mL vancomycin hydrochloride solution for 12 h at room temperature. After incubation, the

samples were carefully removed and gently rinsed with demineralized water to eliminate unbound drug. The Si/APTES and Si/APTES/NPs substrates were treated in the same manner for comparison.

The amount of vancomycin loaded onto each substrate was quantified by measuring the decrease in vancomycin concentration in the loading solution. After loading, the remaining solution (1 mL) was collected and its absorbance at 280 nm was measured using a UV-Vis spectrophotometer. The loaded amount was calculated from the difference between the initial and remaining concentrations. For release experiments, vancomycin-loaded substrates were immersed in 1 mL of 0.01M phosphate-buffered saline (pH 7.0) at 37 °C and gently shaken. Vancomycin was released over 24, 48, and 72 h. At each time point, the release medium was collected and analyzed at 280 nm to determine the vancomycin concentration, as described above.

Characterizations

The surface morphology and nanostructure of the samples were examined using scanning electron microscopy (SEM, JEOL 7800F Prime) equipped with an energy-dispersive X-ray spectroscopy (EDS) detector for elemental analysis. The SEM was operated at an accelerating voltage of 5 kV. EDS mapping and point analysis were conducted to confirm the elemental distribution and composition of the coating materials. X-ray photoelectron spectroscopy (XPS, PHI VersaProbe III) was used to further analyze the surface chemistry. A monochromatic Al Kα X-ray source (1486.6 eV) was applied, and the take-off angle was 90° relative to the surface. The analysis was performed using a 100 μm spot size. The data analysis was performed with CasaXPS software. Raman spectra were collected using a WITec alpha300 R Raman Microscope with 100 × objective, 532 nm laser and 600 g/mm grating. Spectra were acquired from the range 500-3000 cm^{-1} and 0.5 s integration time. The surface wettability was evaluated by measuring the water contact angle in the air using an optical tensiometer (Attension, Biolin Scientific) with approximately 5 μL deionized water droplets. The images were taken within 5 seconds of the droplet being dispensed on the sample. The three independently prepared samples were evaluated for each coating type, and the results are reported as mean ± standard deviation.

Antibacterial assays

The *Staphylococcus aureus* strain (CCUG 35571) was used to evaluate the antibacterial activity of the coatings. Bacteria from an overnight culture in tryptic soy broth (TSB) were diluted 100-fold in fresh medium to a final concentration of 2-5×10^5 colony-forming units (CFU)/mL. The inoculum was applied to the Si/APTES/NPs/VG and incubated at 37 °C under static conditions. After 24 h of biofilm growth, the medium was carefully removed, and the surfaces were washed three times with sterile PBS to eliminate free-floating bacteria without disrupting the biofilms. The biofilms were then stained with 0.1%

crystal violet for 20 min. Excess stain was removed, and the samples were rinsed five times with sterile water, followed by drying at room temperature. To extract the bound stain, 33% acetic acid was added to the samples, which were then agitated vigorously for 30 min. The optical density of the extracted crystal violet was measured at 590 nm. To evaluate the long-term ability of the coatings to inhibit biofilm formation, biofilms were allowed to develop on the coated substrates for up to 72 h. A bacterial inoculum was added onto each sample, followed by incubation at 37 °C. The culture medium was replaced every 24 h to sustain bacterial viability over the extended incubation period. At 24, 48, and 72 h, the adherent biofilms were collected by scraping and homogenizing the cells. The resulting suspensions were serially diluted and plated onto agar plates for viable colony enumeration.

In addition, anti-biofilm activity was assessed by viable cell counts, scanning electron microscopy (SEM), and live/dead staining. For CFU quantification, the inoculum (2-5 × 10^5 CFU/mL) was applied to the Si/APTES/NPs/VG and incubated at 37 °C under static conditions for 24 h. The culture medium was then removed, and irreversibly attached bacteria were detached from the surfaces by sonication (Digital Sonifier, Branson; 10% amplitude, 30 s) in 5 mL of 0.89% NaCl. The collected bacteria were serially diluted (10-fold) and plated on agar plates, which were incubated at 37 °C for 24 h. Colony counts were used to calculate the number of viable bacteria (CFU/sample) according to the dilution factors. Morphological changes of biofilms were examined by SEM. Biofilms formed on the coated surfaces were fixed in 3% glutaraldehyde for 2 h, followed by dehydration in a graded ethanol series (40%, 50%, 60%, 70%, 80%, 90%, and 100%) for 20 min each. The dehydrated samples were dried, sputter-coated with a 15 nm gold layer to prevent charging. For live/dead staining, biofilms were stained with SYTO 9 and potassium iodide using the Live/Dead BacLight Viability Kit (L13152) and imaged with a fluorescence microscope (Axio Imager Z2m, Zeiss, Germany).

Biocompatibility

MG-63 cells were used for the experiment. The cells were maintained at 37 °C in a humidified atmosphere with 5% CO_2 in Minimum Essential Medium Eagle (Thermo Scientific), supplemented with 10% fetal bovine serum (Thermo Scientific). Cells were seeded in 24-well plates at a density of 1 × 10^5 cells per well in 1 mL of medium and cultured for 24 h in the presence of the test samples. After incubation, wells containing the samples were treated with 1× alamarBlue reagent (Thermo Scientific) and incubated for 3 h at 37 °C. Fluorescence was measured using a FLUOStar Omega plate reader, and the results were normalized to the control (untreated silicone) samples. For bright-field imaging, cell morphology was observed on the bottom surface of the well plate, surrounding the substrates. After gentle PBS washing to remove non-adherent cells and debris, images were captured using a VWR VisiScope IT415PH inverted optical microscope equipped with a digital camera. These images served to qualitatively assess cell spreading and viability near the sample area. For SEM imaging on the

substrates, the samples were fixed with 3% glutaraldehyde for 3 h. The fixed samples were then dehydrated in a graded ethanol series (40%, 50%, 60%, 70%, 80%, 90%, and 100%), with each step lasting 10 min. Finally, the samples were air-dried at room temperature for 2 h prior to imaging.

Statistical Analysis

All measurements were performed in triplicate under identical conditions. Data are presented as mean ± standard deviation. Statistical analysis was conducted using one-way analysis of variance (ANOVA) followed by Tukey's post hoc multiple comparison test. Differences between groups were considered statistically significant at *$p<0.05$, **$p < 0.01$, ***$p < 0.001$.

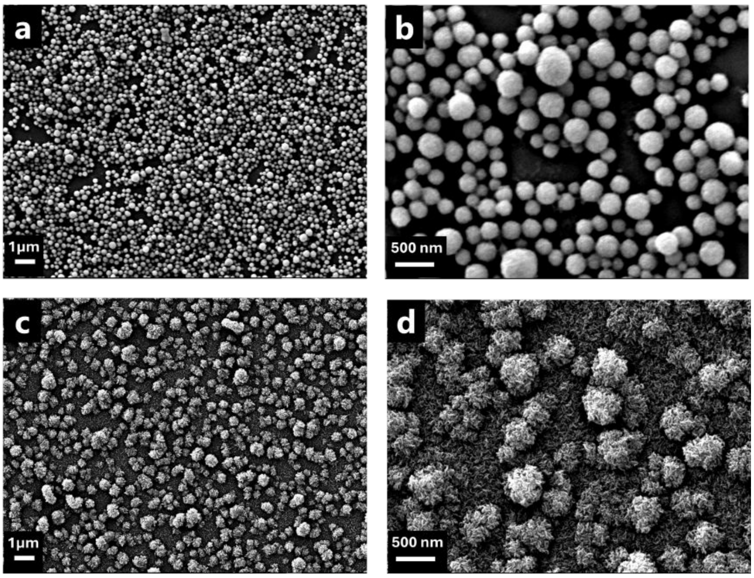

Figure S1. SEM images of Si/APTES/NPs (a, b) and Si/APTES/NPs/VG (c, d).

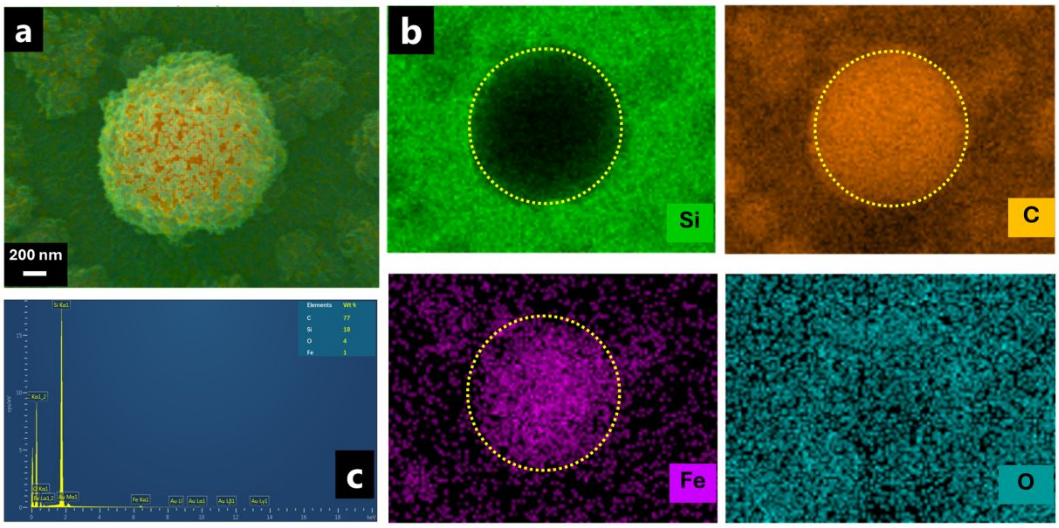

Figure S2. SEM image of Si/APTES/NPs/VG (a); corresponding EDS mapping images of Si, C, Fe, and O (b); and EDS spectrum of Si/APTES/NPs/VG (c).

Table S1. Surface elemental composition of Si/APTES/NPs/VG and Si/APTES/NPs/VG/Van.

Coatings	Element composition （at%）			
	C	O	N	Cl
Si/APTES/NPs/VG	97.54	2.46	-	-
Si/APTES/NPs/VG/Van	87.59	9.77	2.17	0.48

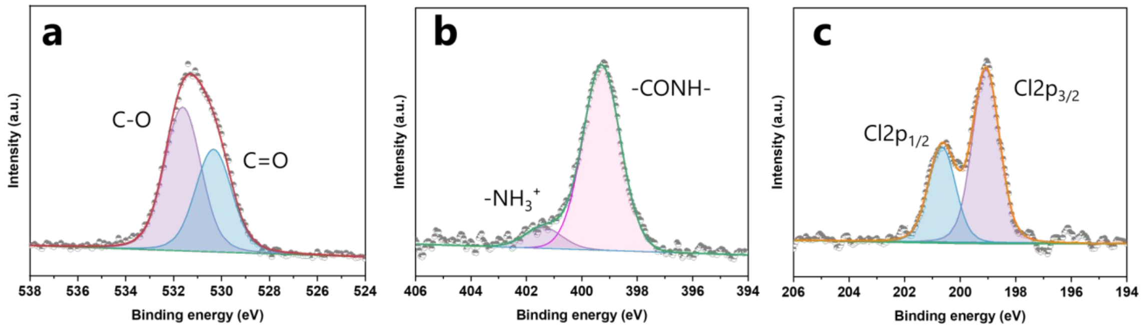

Figure S3. High-resolution O1s (a), N1s (b), and Cl2p (c) spectra for Si/APTES/NPs/VG/Van.

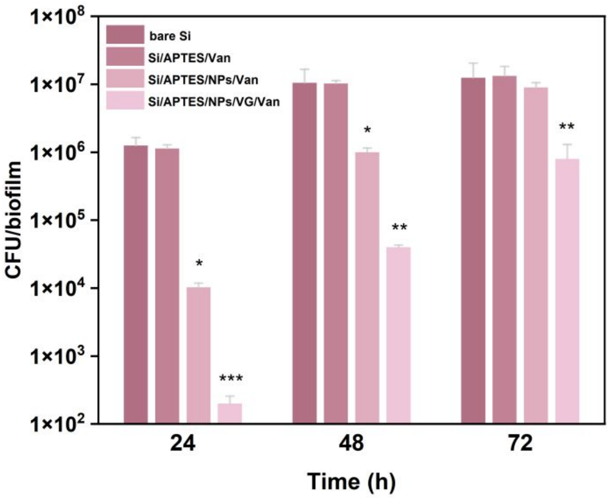

Figure S4. Viability of bacteria in 3 days biofilms grown on bare Si, Si/APTES/Van, Si/APTES/NPs/Van and Si/APTES/NPs/VG/Van. Data represents mean ± standard deviation from 3 independent biological replicates (*p<0.05, **p < 0.01, ***p < 0.001).

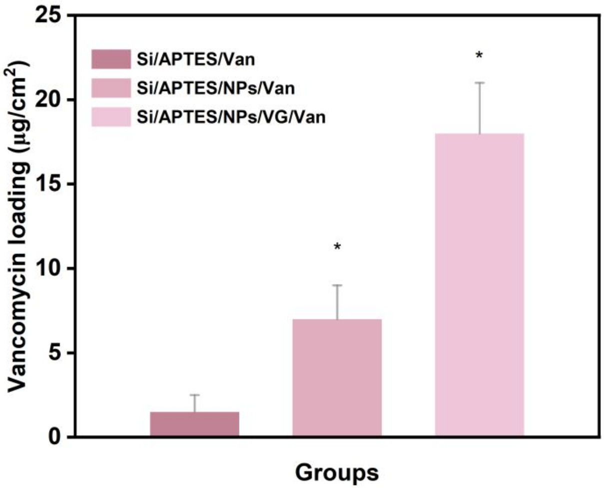

Figure S5. The amount of vancomycin loaded on Si/APTES, Si/APTES/NPs and Si/APTES/NPs/VG. Data represents mean ± standard deviation from 3 independent biological replicates (*p<0.05).

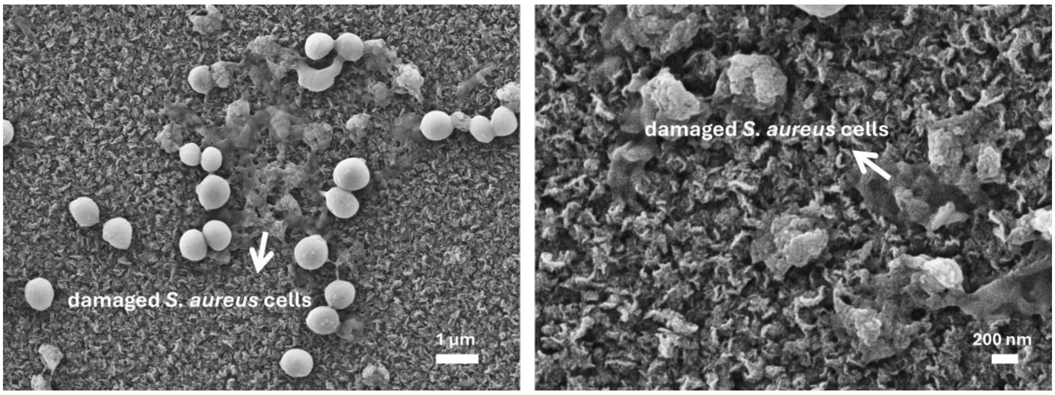

Figure S6. SEM images of *S. aureus* after 24 h contact with VG surfaces.
